# Comparison of antimicrobial activities and resistance mechanisms of eravacycline and tigecycline against clinical *Acinetobacter baumannii* isolates in China

**DOI:** 10.3389/fmicb.2024.1417237

**Published:** 2024-09-24

**Authors:** Xiandi Chen, Yitan Li, Yingzhuo Lin, Yingyi Guo, Guohua He, Xiaohu Wang, Mingzhen Wang, Jianbo Xu, Mingdong Song, Xixi Tan, Chao Zhuo, Zhiwei Lin

**Affiliations:** ^1^Key Laboratory of Respiratory Disease, People’s Hospital of Yangjiang, Yangjiang, China; ^2^Guangzhou Institute of Respiratory Health, First Affiliated Hospital of Guangzhou Medical University, Guangzhou, China

**Keywords:** *Acinetobacter baumannii*, eravacycline, tigecycline, antimicrobial activity, resistance mechanism, ISAba1 insertion

## Abstract

Tigecycline (TGC) is currently used to treat carbapenem-resistant *Acinetobacter baumannii* (CRAB) infections, while eravacycline (ERV), a new-generation tetracycline, holds promise as a novel therapeutic option for these infections. However, differences in resistance mechanism between ERV and TGC against *A. baumannii* remain unclear. This study sought to compare the characteristics and mechanisms of ERV and TGC resistance among clinical *A. baumannii* isolates. A total of 492 isolates, including 253 CRAB and 239 carbapenem-sensitive *A. baumannii* (CSAB) isolates, were collected from hospitalized patients in China. The MICs of ERV and TGC against *A. baumannii* were determined by broth microdilution. Genetic mutations and expressions of *adeB, adeG, adeJ, adeS, adeL*, and *adeN* in resistant strains were examined by PCR and qPCR, respectively. The *in vitro* recombination experiments were used to verify the resistance mechanism of ERV and TGC in *A. baumannii*. The MIC_90_ of ERV in CRAB and CSAB isolates were lower than those of TGC. A total of 24 strains resistant to ERV and/or TGC were categorized into three groups: only ERV-resistant (*n* = 2), both ERV- and TGC-resistant (*n* = 7), and only TGC-resistant (*n* = 15). ST208 (75%, *n* = 18) was a major clone that has disseminated in all three groups. The IS*Aba1* insertion in *adeS* was identified in 66.7% (6/9) of strains in the only ERV-resistant and both ERV- and TGC-resistant groups, while the IS*Aba1* insertion in *adeN* was found in 53.3% (8/15) of strains in the only TGC-resistant group. The *adeABC* and *adeRS* expressions were significantly increased in the only ERV-resistant and both ERV- and TGC-resistant groups, while the *adeABC* and *adeIJK* expressions were significantly increased and *adeN* was significantly decreased in the only TGC-resistant group. Expression of *adeS* with the IS*Aba1* insertion in ERV- and TGC-sensitive strains significantly increased the ERV and TGC MICs and upregulated *adeABC* and *adeRS* expressions. Complementation of the wildtype *adeN* in TGC-resistant strains with the IS*Aba1* insertion in *adeN* restored TGC sensitivity and significantly downregulated *adeIJK* expression. In conclusion, our data illustrates that ERV is more effective against *A. baumannii* clinical isolates than TGC. ERV resistance is correlated with the IS*Aba1* insertion in *adeS*, while TGC resistance is associated with the IS*Aba1* insertion in *adeN* or *adeS* in *A. baumannii.*

## 1 Introduction

*Acinetobacter baumannii* is a serious hospital-acquired pathogen associated with wounds, urinary tract infections, bloodstream infections, pneumonia, and meningitis (Gerson et al., 2018). The attributable mortality of patients with *A. baumannii* ventilator-associated pneumonia and bloodstream infections is as high as 54% in the intensive care unit (ICU) (Alosaimy et al., 2022). In recent years, the widespread use of carbapenem antibiotics has caused the emergence and spread of carbapenem-resistant *Acinetobacter baumannii* (CRAB), posing a significant threat to global public health (Liu et al., 2024). Given the widespread resistance of CRAB to almost all commonly used antibiotics, available treatment options are extremely limited, with none able to effectively reduce mortality (O'Donnell et al., 2021). The antibiotics currently used to treat CRAB infection include tigecycline (TGC), polymyxins, and the *β*-lactamase inhibitor, sulbactam (Serapide et al., 2024). However, the antibiotics described above have some limitations in clinical application. Polymyxins can cause nephrotoxicity, which substantially narrows the therapeutic window (LaPlante et al., 2018). TGC suffers from pharmacokinetic issues, such as low plasma levels, limiting its clinical use (Isler et al., 2019). Sulbactam used as monotherapy has a high resistance rate, it is usually used in combination therapies (Deveci et al., 2012). Therefore, novel antimicrobials have been explored for the treatment of CRAB infections in recent years, including cefiderocol, new tetracycline analogues (eravacycline and TP-6076), and new *β*-lactamase inhibitor combinations (Sulbactam-ETX2514, meropenem- WCK 4234, and LN-1-255-carbapenem) (Isler et al., 2019). Among these novel antibiotics, ERV demonstrates superior activity against CRAB and holds promising prospects for the treatment of CRAB infections (Isler et al., 2019).

Eravacycline (ERV) is a novel fluorocycline antibacterial agent that is structurally similar to TGC with two modifications to the D-ring of the tetracycline core. Those modifications greatly improved its bioavailability and made it exhibiting more potent *in vitro* activity against CRAB than TGC (Zhanel et al., 2016). ERV was approved by the Food and Drug Administration (FDA) for treating complicated intra-abdominal infections in 2018 (Alosaimy et al., 2020). It has broad-spectrum activity against both gram-positive and gram-negative bacteria, including some antibiotic-resistant strains, such as multidrug-resistant *A. baumannii*, vancomycin-resistant *Enterococcus*, methicillin-resistant *Staphylococcus aureus*, and carbapenem-resistant *Enterobacteriaceae* (Sutcliffe et al., 2013; Monogue et al., 2016; Li Y. et al., 2022). As a result, ERV is currently in development as an intravenous (i.v.) and oral medication for treating serious infections caused by antibiotic-resistant bacteria (Zhanel et al., 2016). Notably, ERV is four times as effective as TGC against multidrug-resistant *A. baumannii* (Zhanel et al., 2016). Patients with complicated intra-abdominal CRAB infections achieved a 100% clinical and microbiologic cure following ERV treatment (Poulakou et al., 2018). Thus, ERV is a promising option for treating CRAB infections.

TGC resistance is primarily associated with overexpression of RND efflux pumps, including AdeABC, AdeFGH, and AdeIJK, that are controlled by the transcriptional regulators AdeRS, AdeL, and AdeN, respectively (Lucaßen et al., 2021b). Mutations or insertions in these regulatory genes can also promote TGC resistance by increasing efflux pump expression (Hornsey et al., 2010). In addition, other genes, including the ribosomal S10 protein-encoding gene *rpsJ*, MFS efflux pumps *tet(A)* and its regulatory factors *tetR*, plasmid-mediated *tet*(X5), antibiotic targets of action *rrf*, *rpoB*, and global regulator *soxR*, were also reported to be associated with TGC resistance (Beabout et al., 2015; Wang et al., 2019; Hua et al., 2021; Sun et al., 2023). Shi et al. showed that a deletion mutation in the *adeS* gene of induced resistant strains correlates with high AdeABC expression and increased ERV resistance (Shi et al., 2020). However, the mechanism of *A. baumannii* resistance to ERV and the differences between the ERV and TGC resistance mechanisms require additional clarification.

In the present study, we sought to compare the traits and resistance mechanisms in ERV- and TGC-resistant *A. baumanni* isolates from China. The *in vitro* antimicrobial activity of ERV, TGC, and other clinical agents was examined; the presence of clonality and carbapenemase resistance genes were analyzed. Furthermore, efflux pump inhibition assays, molecular sequencing, quantitative real-time PCR (qRT-PCR) and functional verification experiments were performed to explore various resistance mechanism of ERV and TGC in *A. baumanni*.

## 2 Materials and methods

### 2.1 Bacterial isolates, antibiotics, and growth conditions

A total of 492 non-duplicate clinical *A. baumannii* isolates, including 253 CRAB and 239 CSAB, were collected from patients who were admitted to Yangjiang People’s Hospital, a 2,000-bed tertiary hospital in Guangdong Province of China from January 2018 to December 2022. The *A. baumannii* isolates were identified using a VITEK 2 compact system (BioMérieux, Marcy l’Etoile, France) and further confirmed by VITEK mass spectrometry (BioMérieux, Marcy l’Etoile, France). All strains were cultured in Mueller-Hinton (MH) (Oxoid, Basingstoke, UK) or Luria-Bertani (LB) broth (Oxoid, Basingstoke, UK) at 37°C. *Escherichia coli* ATCC25922 was used as a quality control strain. All procedures performed were approved by the Ethical Committee of Yangjiang People’s Hospital and were in accordance with the 1964 Helsinki Declaration and its later amendments. ERV and TGC were obtained from MedChem Express (Shanghai, China), and the other antimicrobials were purchased from Meilunbio (Dalian, China).

### 2.2 Antimicrobial susceptibility testing

The minimum inhibitory concentration (MIC) of ERV, TGC, polymyxin B, meropenem, imipenem, levofloxacin, gentamicin, cefepime, amikacin, ceftazidime, minocycline, piperacillin/tazobactam and ampicillin/sulbactam against *A. baumannii* isolates were determined using broth microdilution method according to the guidelines of Clinical and Laboratory Standards Institute (CLSI) M100-S34 (Clinical and Laboratory Standards Institute, 2024). Since the *A. baumannii* MIC breakpoints for ERV and TGC have not yet been established by CLSI and the FDA, the MIC values were categorized into three levels according to prior studies (Marchaim et al., 2014; Abdallah et al., 2015): ≤2 mg/L (susceptible), 4 mg/L (intermediate), and ≥ 8 mg/L (resistant). The susceptibility results for other antibiotics were determined based on CLSI breakpoints. In this study, CRAB strains were defined as those with an imipenem and/or meropenem MIC ≥8 mg/L, while CSAB strains were defined as those with imipenem and meropenem MICs ≤4 mg/L (Chen et al., 2023).

### 2.3 Polymerase chain reaction for multilocus sequence typing, carbapenemase, and genetic mutations in the resistance genes

Total DNA from ERV- and/or TGC-resistant strains were extracted using Lysis Buffer for Microorganisms to Direct PCR (Takara Bio Inc., Japan). PCR was performed using PCR Mastermix (Thermo Fisher Scientific, Waltham, MA) according to the manufacturer’s instructions. Multilocus sequence typing (MLST) of the strains was determined by PCR and sequence alignment, as described previously (Li T. et al., 2022). The carbapenemase resistance genes, *bla*_OXA_, *bla*_KPC_, *bla*_IMP_, *bla*_VIM_, *bla*_SIM_, and *bla*_NDM-1_ were detected by PCR (Ramirez et al., 2020). Genetic mutations of the RND efflux pump regulatory genes, including *adeR, adeS, adeL, adeN*, and other genes associated with TGC resistance, including *rpsJ*, *tet(A)*, *tetR*, *tet*(X5), *rrf, rpoB*, and *soxR* (Beabout et al., 2015; Wang et al., 2019; Hua et al., 2021; Sun et al., 2023), were amplified by PCR and sequenced. Mutations in the above-mentioned genes in ERV- and TGC-resistant *A. baumannii* were identified by comparing them with the *A. baumannii* strain ATCC BAA1605 (GenBank accession number: CP058625) (Ten et al., 2021). The primers used for PCR are listed in Supplementary Tables S1–S3.

### 2.4 Efflux pump inhibition assays

The efflux pump activity in ERV- and/or TGC-resistant *A. baumannii* isolates was detected using the efflux pump inhibitors (EPIs), Phe-Arg-*β*-naphthylamide (PaβN; MCE, Shanghai, China) and carbonyl cyanide m-chlorophenylhydrazone (CCCP; MCE, Shanghai, China). ERV and TGC MICs were determined using the agar dilution method in the presence and absence of PAβN (50 mg/L) or CCCP (16 mg/L) in ERV- and TGC-resistant strains (Jo and Ko, 2021). Significant inhibition of the efflux pumps was determined by the reduction of MIC to a quarter (or more) of their baseline values in the presence of EPI (Zheng et al., 2018).

### 2.5 Quantitative real-time PCR analysis

Expression levels of the RND efflux pump genes (*adeB, adeG,* and *adeJ*) and their regulators (*adeS, adeL*, and *adeN*) were determined in ERV- and/or TGC-resistant *A. baumannii* using qRT-PCR. Ten ERV- and TGC-susceptible strains selected from similar sources of the resistant isolates were used as sensitive control strains. Expressions of the target genes in the three groups of resistant strains were compared with their expressions in the sensitive strains. Since *adeA, adeB*, and *adeC* are located within the same operon, *adeB* was considered representative of *adeABC* efflux pump expression in this study (Yang et al., 2019). The other genes shared the same principle. In brief, overnight cultures of the bacterial strains were diluted 1:100 in 10 mL of LB broth and incubated at 37°C with 220 rpm shaking until the growth had reached a logarithmic phase. Total bacterial RNA was extracted with RNA Extraction Kit (Vazyme, Nanjing, China), and cDNA was synthesized with a HiScriptIII-RT SuperMix for qRT-PCR kit (Vazyme, Nanjing, China). Finally, qRT-PCR was performed using a ChamQ Universal SYBR qRT-PCR Master Mix (Vazyme, Nanjing, China) in a LightCycler480II system (Roche, Basel, Switzerland), with the following parameters: 1 cycle at 95°C for 30 s, followed by 45 cycles of 95°C for 10 s and 60°C for 30 s (Li et al., 2024). Each sample was run in triplicate. Expressions of the target genes were normalized relative to the internal control gene, *rpoB* (Jo and Ko, 2021). Threshold cycle (Ct) numbers were confirmed with qRT-PCR system software, and the data were analyzed using the 2^−ΔΔCt^ method. The CRAB-20 isolate, containing all the efflux pumps involved in this study and sensitive to both ERV and TGC, was used as the reference strain (expression = 1). The primers used for qRT-PCR are listed in Supplementary Table S4.

### 2.6 Functional verification of efflux pump gene mutations

Previous study has reported that IS*Aba1* is inserted within the *adeS* gene. The Pout promoter of the IS*Aba1* sequence will then replace the native promoter and drive the translation of a truncated *adeS* gene. The truncated AdeS protein was able to activate AdeR and then enhance the *adeABC* gene expression. Overexpression of AdeABC ultimately confers resistance to TGC in *A. baumannii* (Sun et al., 2012). To confirm the role of the IS*Aba1* insertion in *adeS* on ERV resistance, *adeS* with the IS*Aba1* insertion was amplified from the CRAB-42 isolate by PCR and integrated into the expression plasmid, pACYC184, with *Bam*HI and *Cla*I cloning sites (Guo et al., 2021). Positive clones were screened with chloramphenicol and verified by PCR and sequencing. The resulting constructs (named: padeS-ISAbal) were separately transformed into five strains both susceptible to ERV and TGC by electroporation and further identified by PCR and sequencing. The pACYC184 and pACYC184 inserted with wildtype *adeS* (named: padeS-WT) were separately introduced into the same strains as a control. Previous research has shown that the insertion of IS*Aba1* within the *adeN* gene led to the disruption of *adeN*, which caused the loss of *adeN* repressive function on *adeIJK* gene and ultimately resulted in the overexpression of AdeIJK (Lucaßen et al., 2021a). To assess the impact of the IS*Aba1* insertion in *adeN* on TGC resistance, wildtype *adeN* with its own promoter region was amplified from the CRAB-86 isolate and cloned into the pACYC184 plasmid as described above. The complementary plasmid (named: padeN-WT) was verified by PCR and sequencing and transformed into five TGC-resistant strains with IS*Aba1* inserted in *adeN* by electroporation. The pACYC184 was transferred into the same strains as a control. The susceptibility of ERV and TGC was then evaluated in the constructed strains using broth microdilution method. Expressions of *adeB*, *adeS*, or *adeJ* in the constructed strains were further confirmed by qRT-PCR. The strains and primers used for the functional verification experiment are listed in Supplementary Tables S5, S6.

### 2.7 Statistical analysis

The Student’s *t*-test was used for continuous variables. All tests were performed using IBM SPSS Statistics (version 22.0; IBM, Chicago, United States). *p*-values <0.05 are regarded as statistically significant.

## 3 Results

### 3.1 Characteristics and *in vitro* antimicrobial susceptibility of the bacterial strains

A total of 492 clinical *A. baumannii* isolates, including 253 isolates of CRAB and 239 isolates of CSAB, were analyzed in this study. The *A. baumannii* strains were isolated from sputum (44.5%), wound secretions (24.6%), urine (13.6%), blood (8.9%), and other sources (including bronchoalveolar lavage fluid, ascites, abscess, bile, cerebrospinal fluid, and pleural effusion) (8.3%) (Supplementary Table S7). CRAB was primarily found in sputum and wound secretions, while CSAB isolates were relatively dispersed. The susceptibility of 492 *A. baumannii* isolates to ERV, TGC, and other antibiotics is summarized in Table 1. The results show that 2.4 and 5.9% of the CRAB isolates were resistant to ERV and TGC, respectively, while only 1.3 and 2.9% of the CSAB isolates were resistant to these antibiotics, respectively. The MIC_90_ of ERV for CRAB and CSAB was 1 mg/L and 0.5 mg/L, respectively, which was lower than the MIC_90_ of TGC for CRAB (2 mg/L) and CSAB (1 mg/L), suggesting that ERV is more effective against *A. baumannii* than TGC. Additionally, CRAB exhibited resistance to other commonly used antibiotics, including levofloxacin, gentamicin, cefepime, amikacin, ceftazidime, piperacillin/tazobactam, and ampicillin/sulbactam; however, CSAB was sensitive to these drugs.

**Table 1:** Antimicrobial susceptibility of 492 *A. baumannii* clinical isolates.

Antibiotics	Breakpoints (mg/L)^a^			MIC Range (mg/L)	MIC_50_ (mg/L)	MIC_90_ (mg/L)	Resistant (*n*,%)
	S	I	R				
**CRAB** ^**b**^ **(*n* = 253)**							
Eravacycline	≤2	4	≥8	0.12–16	0.5	1	6, 2.4%
Tigecycline	≤2	4	≥8	0.5–32	1	2	15, 5.9%
Polymyxin B	≤2	–	≥4	0.5–>16	1	2	5, 2.0%
Levofloxacin	≤2	4	≥8	4–>128	>128	>128	243, 96.0%
Gentamicin	≤4	8	≥16	1–>128	>128	>128	249, 98.4%
Cefepime	≤8	16	≥32	16–>128	>128	>128	250, 98.8%
Amikacin	≤16	32	≥64	4–>128	>128	>128	250, 98.8%
Ceftazidime	≤8	16	≥32	16–>128	>128	>128	248, 98.0%
Meropenem	≤2	4	≥8	8–>128	>128	>128	253, 100.0%
Minocycline	≤4	8	≥16	1–128	4	8	46, 18.2%
Piperacillin/Tazobactam	≤16/4	32/4–64/4	≥128/4	16/4 ~ >128/4	>128/4	>128/4	250, 98.8%
Ampicillin/Sulbactam	≤8/4	16/8	≥32/16	16/8 ~ >128/64	>128/64	>128/64	248, 98.0%
**CSAB (*n* = 239)**							
Eravacycline	≤2	4	≥8	0.03–8	0.12	0.5	3, 1.3%
Tigecycline	≤2	4	≥8	0.25–16	0.5	1	7, 2.9%
Polymyxin B	≤2	–	≥4	0.25–8	1	2	3, 1.3%
Levofloxacin	≤2	4	≥8	0.06–32	0.12	1	5, 2.1%
Gentamicin	≤4	8	≥16	0.25–32	0.5	1	8, 3.3%
Cefepime	≤8	16	≥32	0.5–64	1	2	3, 1.3%
Amikacin	≤16	32	≥64	0.12–4	0.5	1	0, 0%
Ceftazidime	≤8	16	≥32	0.12–128	2	4	5, 2.1%
Meropenem	≤2	4	≥8	0.03–4	0.25	0.5	0, 0%
Minocycline	≤4	8	≥16	0.12–32	0.5	2	17, 7.1%
Piperacillin/Tazobactam	≤16/4	32/4–64/4	≥128/4	1/4–>128/4	4/4	16/4	8, 3.3%
Ampicillin/Sulbactam	≤8/4	16/8	≥32/16	1/0.5–>128/64	2/1	4/2	5, 2.1%

a S, susceptible; I, intermediate; R, resistant. As *A. baumannii* MIC breakpoints for eravacycline and tigecycline have not yet been established by CLSI and FDA, this study categorized the MIC values into three levels according to prior studies (Marchaim et al., 2014; Abdallah et al., 2015): ≤2 mg/L (susceptible), 4 mg/L (intermediate), and ≥ 8 mg/L (resistant).

b CRAB, Carbapenem-resistant A. baumannii; CSAB, carbapenem-susceptible A. baumannii.

### 3.2 Comparison of the characteristics of ERV- and/or TGC-resistant isolates

A total of 24 strains, identified as resistant to ERV and/or TGC, were categorized into three groups: only ERV-resistant (*n* = 2), both ERV- and TGC-resistant (*n* = 7), and only TGC-resistant (*n* = 15) (Table 2). The source, clinical department, MLST, and infected populations of each group had similar characteristics (Table 2). The 24 resistant strains came from various sources, including sputum (*n* = 11, 45.8%), wound secretions (*n* = 8, 33.3%), and others (*n* = 5, 20.8%). They were primarily obtained from patients in the ICU (*n* = 11, 45.8%) and individuals >60 years of age (*n* = 17, 71%). Three patients infected with a resistant CRAB strain had a fatal clinical outcome (*n* = 3, 12.5%). Most (*n* = 18, 75%) of the strains belonged to ST208, and all resistant CRAB isolates contained *bla*_OXA-23_ carbapenemases. However, there were clear differences in the ERV and TGC MICs of the three groups. The ERV MICs were 2–4x higher than the TGC MICs in the only ERV-resistant group; the TGC MICs were 1–4x higher than the ERV MICs in the both ERV- and TGC-resistant group. Meanwhile, the TGC MICs were 4–64x higher than the ERV MICs in the TGC-resistant group, suggesting that the antimicrobial activity of ERV is superior to TGC.

**Table 2:** Comparison of the characteristics of ERV- and/or TGC-resistant clinical isolates.

Strains (*n* = 24)	ERV MIC (mg/L)	TGC MIC (mg/L)	Gender	Age (year)	Source	Department	Outcomes	MLST	Resistance gene
Only ERV-resistant group (*n* = 2)									
CRAB-18^a^	8	2	Female	50	Wound secretion	Intensive Care Unit	Cure	208	*bla*_OXA-23_, *bla*_OXA-24_, *bla*_OXA-51_
CSAB-211	8	4	Male	31	Wound secretion	Department of Trauma and Orthopedics	Cure	208	*bla*_OXA-51_
Both ERV- and TGC-resistant group (*n* = 7)									
CRAB-42	8	16	Male	60	Sputum	Intensive Care Unit	Cure	208	*bla*_OXA-23_, *bla*_OXA-24_, *bla*_OXA-51_
CRAB-105	8	16	Male	79	Wound secretion	Department of General Family Medicine	Cure	208	*bla*_OXA-23_, *bla*_OXA-58_, *bla*_OXA-51_
CRAB-133	16	32	Male	88	Sputum	Intensive Care Unit	Cure	208	*bla*_OXA-23_, *bla*_OXA-24_, *bla*_OXA-51_
CRAB-177	8	32	Female	78	Wound secretion	Department of Neurology	Cure	1486	*bla*_OXA-23_, *bla*_NDM-1_, *bla*_OXA-51_
CRAB-194	8	16	Male	75	Sputum	Intensive Care Unit	Death	208	*bla*_OXA-23_, *bla*_VIM_, *bla*_OXA-51_
CSAB-45	8	8	Male	65	Blood	Department of Gastrointestinal Surgery	Cure	208	*bla*_OXA-51_
CSAB-107	8	16	Male	47	Sputum	Intensive Care Unit	Cure	191	*bla*_OXA-24,_ *bla*_OXA-51_
Only TGC-resistant group (*n* = 15)									
CRAB-35	2	16	Male	81	Wound secretion	Intensive Care Unit	Cure	208	*bla*_OXA-23_, *bla*_OXA-24、_*bla*_OXA-51_
CRAB-61	2	32	Male	68	Wound secretion	Department of Neurosurgery	Cure	191	*bla*_OXA-23_, *bla*_OXA-24、_*bla*_OXA-51_
CRAB-86	2	8	Male	63	Sputum	Department of Rheumatology	Cure	208	*bla*_OXA-23_, *bla*_OXA-51_
CRAB-110	0.25	8	Male	44	Cerebrospinal fluid	Department of Neurosurgery	Cure	208	*bla*_OXA-23_, *bla*_OXA-24、_*bla*_OXA-51_
CRAB-123	1	16	Male	78	Sputum	Intensive Care Unit	Cure	208	*bla*_OXA-23_, *bla*_OXA-24、_*bla*_OXA-51_
CRAB-145	0.5	8	Female	62	Urine	Department of Neurology	Cure	1849	*bla*_OXA-23_, *bla*_OXA-24、_*bla*_OXA-51_
CRAB-158	1	16	Male	68	Sputum	Intensive Care Unit	Cure	208	*bla*_OXA-23_, *bla*_OXA-51_
CRAB-161	0.5	16	Male	82	Sputum	Intensive Care Unit	Death	208	*bla*_OXA-23_, *bla*_OXA-24、_*bla*_OXA-51_
CRAB-167	0.12	8	Male	77	Sputum	Intensive Care Unit	Death	208	*bla*_OXA-23_, *bla*_OXA-24、_*bla*_OXA-51_
CRAB-185	2	16	Male	48	Sputum	Intensive Care Unit	Cure	208	*bla*_OXA-23_, *bla*_VIM_, *bla*_OXA-51_
CSAB-79	0.12	8	Male	46	Blood	Department of Blood Purification	Cure	2211	*bla*_OXA-24_, *bla*_OXA-51_
CSAB-93	2	8	Male	69	Sputum	Department of Oncology	Cure	451	*bla*_OXA-51_
CSAB-166	2	8	Male	68	Wound secretion	Department of Burn Plastic Surgery	Cure	208	*bla*_OXA-51_
CSAB-172	0.12	8	Male	71	Wound secretion	Department of Dermatology	Cure	208	*bla*_OXA-51_
CSAB-199	1	16	Male	56	Pleural effusion	Cardio-thoracic Surgery	Cure	208	*bla*_OXA-24_, *bla*_OXA-51_

a CRAB: Carbapenem-resistant *A. baumannii*, CSAB: Carbapenem-susceptible *A. baumannii*.

The susceptibilities of other commonly used antibiotics in the three groups of resistant strains are summarized in Supplementary Table S8. The CRAB isolates that were resistant to ERV and/or TGC exhibited high resistance to levofloxacin, minocycline, amikacin, gentamicin, doxycycline, and cefepime, but still showed sensitivity to polymyxin B. The CSAB strains resistant to ERV and/or TGC only exhibited cross-resistance with minocycline and doxycycline, while remaining sensitive to other commonly used antibiotics.

### 3.3 Effect of the efflux pump inhibitor on the ERV- and/or TGC-resistant isolates

To investigate the potential role of the efflux pump in ERV- and/or TGC-resistant *A. baumannii* strains, the effect of EPIs on the ERV and TGC MICs of resistant strains was assessed. The addition of two EPIs to the only ERV-resistant group significantly reduced the ERV MIC by 4–32x but did not have a significant effect on the TGC MIC (Table 3). The addition of two EPIs to both ERV- and TGC-resistant group significantly reduced the MICs of both antibiotics. After adding PaβN, the ERV and TGC MICs decreased by ≥4x in 100% (*n* = 7) or 85.7% (*n* = 6) of strains, respectively, and after adding CCCP, the ERV and TGC MICs significantly decreased in 71.4% (*n* = 5) or 57.1% (*n* = 4) of strains, respectively. In the only TGC-resistant group, the TGC MIC decreased by ≥4x in the presence of PaβN or CCCP in 77.3% (*n* = 11) or 66.7% (*n* = 10) of the resistant isolates, while the ERV MIC showed no significant change. These results illustrated that EPIs can significantly reduce the ERV and TGC MICs of resistant strains, suggesting that resistance to these antibiotics may be related to the differential expression of efflux pumps.

**Table 3:** MICs of ERV or TGC against the resistant isolates in the absence or presence of efflux pump inhibitors.

Isolate	MLST	MIC (mg/L)									
		ERV	ERV +PaβN^a^	Fold change^b^	ERV+CCCP	Fold change	TGC	TGC+PaβN	Fold change^c^	TGC+CCCP	Fold change
Only ERV-resistant group (*n* = 2)											
CRAB-18	208	8	0.5	16^d^	1	8	2	1	2	2	1
CSAB-211	208	8	0.25	32	2	4	2	0.5	4	1	2
Both ERV- and TGC-resistant group (*n* = 7)											
CRAB-42	208	8	1	8	2	4	16	1	16	2	8
CRAB-105	208	8	2	4	4	2	16	4	4	8	2
CRAB-133	208	16	1	16	0.5	32	32	1	32	2	16
CRAB-177	1486	8	0.5	16	0.5	16	32	2	16	2	16
CRAB-194	208	8	2	4	4	2	16	8	2	8	2
CSAB-45	208	8	1	8	0.5	16	8	1	8	2	4
CSAB-107	191	8	2	4	2	4	16	4	4	8	2
Only TGC-resistant group (*n* = 15)											
CRAB-35	208	2	1	2	1	2	16	4	4	2	8
CRAB-61	191	2	0.5	4	0.25	8	32	4	8	8	4
CRAB-86	208	2	1	2	2	1	8	8	1	4	2
CRAB-110	208	0.25	0.12	2	0.25	1	8	0.5	16	2	4
CRAB-123	208	1	1	1	1	1	16	8	2	8	2
CRAB-145	1849	0.5	0.5	1	0.25	2	8	0.12	64	1	8
CRAB-158	208	1	0.5	2	0.5	2	16	1	16	4	4
CRAB-161	208	0.5	0.25	2	0.5	1	16	1	16	2	8
CRAB-167	208	0.12	0.12	1	0.12	1	8	1	8	2	4
CRAB-185	208	2	0.5	4	0.5	4	16	4	4	8	2
CSAB-79	2211	0.12	0.12	1	0.12	1	8	1	8	1	8
CSAB-93	451	2	1	2	2	1	8	0.5	16	2	4
CSAB-166	208	2	0.5	4	1	2	8	1	8	2	4
CSAB-172	208	0.12	0.06	2	0.12	1	8	4	2	8	1
CSAB-199	208	1	0.5	2	1	1	16	16	1	8	2

a PaβN: Phe-Arg-β-naphthylamide (50 mg/L); CCCP: carbonyl cyanide m-chlorophenylhydrazone (16 mg/L).

b The fold change means the ratio of ERV MIC and (ERV + EPI) MIC.

c The fold change means the ratio of TGC MIC and (TGC + EPI) MIC.

d Efflux inhibition was defined as a four-fold decrease in the MICs from their original values in the presence of EPI (Tambat et al., 2022).

### 3.4 Resistance mechanisms of ERV- and/or TGC-resistant strains

The results described above suggest that efflux pumps may be involved in ERV and TGC resistance. To further evaluate this, PCR and sequencing were used to identify mutations in the regulator genes of the RND efflux pump. An IS*Aba1* insertion in *adeS* was detected in both strains from the only ERV-resistant group, while no mutations were detected in other genes. Four strains (57.1%, 4/7) in both ERV- and TGC-resistant group had an IS*Aba1* insertion in *adeS*, and one had a Q91K mutation in *adeN*. Meanwhile, eight strains (53.3%, 8/15) in the only TGC-resistant group had an IS*Aba1* insertion mutation in *adeN*, one had a double point mutation (T18R and A153D) in *adeN*, one had a double point mutation (L29R and K131N) in *adeS*, and two had mutations in *adeR*, V14G and A91V, respectively (Table 4). In addition, we further assessed the effect of various resistance mechanisms other than RND-type efflux pumps on ERV and TGC resistance, including *rpsJ*, *tet(A), tetR*, *soxR*, *tet*(X5), *rrf,* and *rpoB*. The results showed that a N27P mutation of TetR was detected in CRAB-86 from the only TGC-resistant group, a M247S mutation of Tet(A) in CRAB-194 and a V57I mutation of *rpsJ* in CRAB-105 were detected from both ERV- and TGC-resistant group, while no mutations were detected in other genes. Moreover, *tet*(X5) was not identified in all the 24 strains (Supplementary Table S9).

**Table 4:** Mutations of efflux pump regulators in ERV- and/or TGC-resistant isolates.

Strains (*n* = 24)	Mutation			
	*adeR*	*adeS*	*adeL*	*adeN*
Only ERV-resistant group (*n* = 2)				
CRAB-18	W^a^	*ISAba1*	W	W
CSAB-211	W	*ISAba1*	W	W
Both ERV- and TGC-resistant group (*n* = 7)				
CRAB-42	W	*ISAba1*	W	W
CRAB-105	W	W	W	W
CRAB-133	W	*ISAba1*	W	W
CRAB-177	W	*ISAba1*	W	Q91K
CRAB-194	W	W	W	W
CSAB-45	W	*ISAba1*	W	W
CSAB-107	W	W	W	W
Only TGC-resistant group (*n* = 15)				
CRAB-35	W	W	W	*ISAba1*
CRAB-61	V14G	W	W	*ISAba1*
CRAB-86	W	W	W	W
CRAB-110	W	W	W	T18R, A153D
CRAB-123	W	W	W	W
CRAB-145	W	W	W	*ISAba1*
CRAB-158	W	L29R, K131N	W	*ISAba1*
CRAB-161	A91V	W	W	W
CRAB-167	W	W	W	*ISAba1*
CRAB-185	W	W	W	W
CSAB-79	W	W	W	*ISAba1*
CSAB-93	W	W	W	*ISAba1*
CSAB-166	W	W	W	*ISAba1*
CSAB-172	W	W	W	W
CSAB-199	W	W	W	W

a “W” indicates that no mutations were detected in the gene.

**Figure 1:**
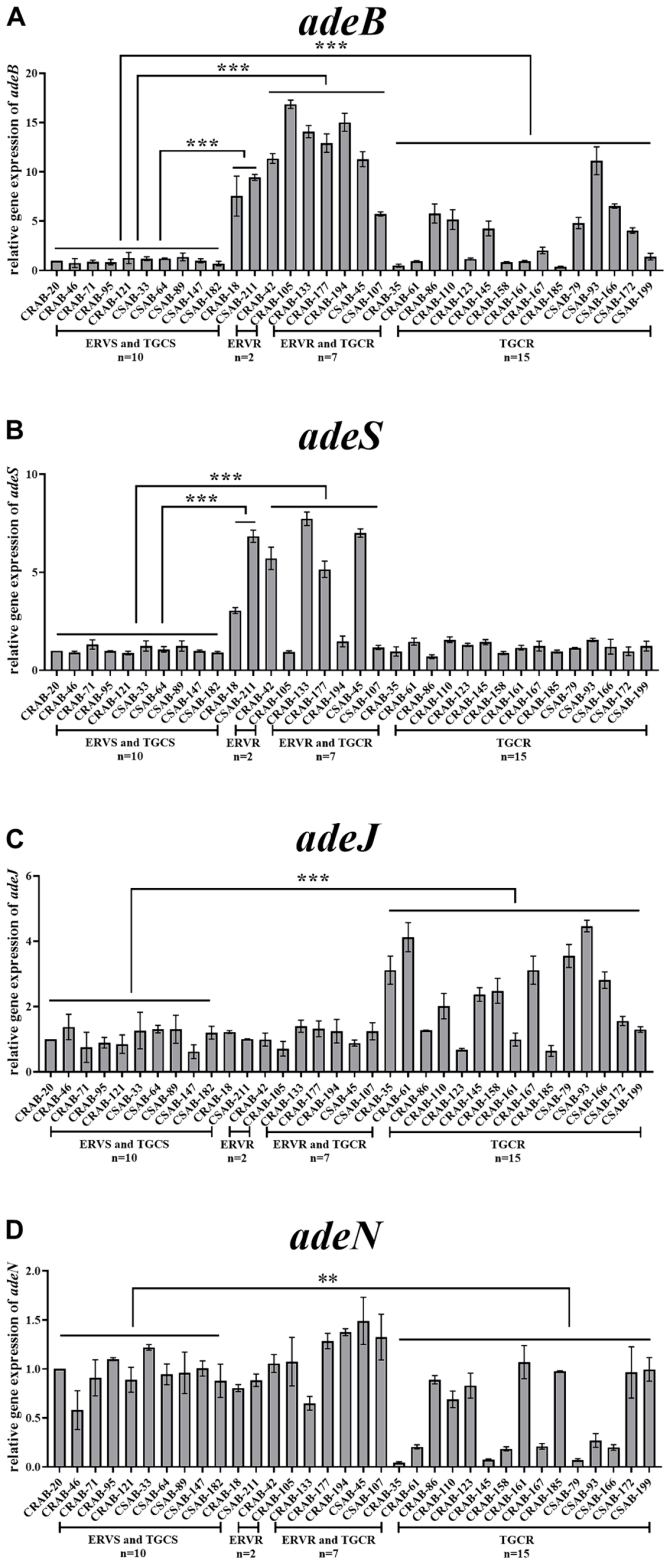
Comparison of the relative expressions of efflux pump genes in ERV- and/or TGC-resistant isolates. Relative expression of *adeB* **(A)**, *adeS* **(B)**, *adeJ* **(C)**, and *adeN* **(D)** were assessed by qRT-PCR analysis. The housekeeping gene, *rpoB,* was used as the endogenous reference gene. CRAB-20 was used as the reference strain (expression = 1.0). All qRT-PCRs were carried out in triplicate. ∗∗∗*p* < 0.01, ∗∗*p* < 0.05. ERVS and TGCS: strains sensitive to both ERV and TGC; ERVR: strains only resistant to ERV; ERVR and TGCR: strains resistant to both ERV and TGC; TGCR: strains only resistant to TGC.

Relative expressions of the RND efflux pump genes were then compared between the resistant and sensitive strains. The expressions of a*deB* and *adeS* were significantly higher in both strains from the only ERV-resistant group than in the sensitive strains. No significant differences were found in the expression of *adeG, adeL, adeJ*, and *adeN*. The expressions of *adeB* and *adeS* were significantly higher in 100% (*n* = 7) and 57.1% (*n* = 4) of strains from the both ERV- and TGC-resistant group than in the sensitive strains, respectively, but no significant differences were found in the expression of other efflux pump genes. The expressions of *adeB* and *adeJ* were significantly higher in 46.7% (*n* = 7) and 60.0% (*n* = 9) of strains from the only TGC-resistant group, respectively, while the expression of *adeN* was significantly lower in 53.3% (*n* = 8) of the strains. The expression of *adeS, adeG*, and *adeL* did not change significantly (Figures 1A–D, Supplementary Figures S1A,B).

### 3.5 Validation of the role of the IS*Aba1* insertion in *adeS* or *adeN* on ERV and/or TGC antibiotic resistance

**Figure 2:**
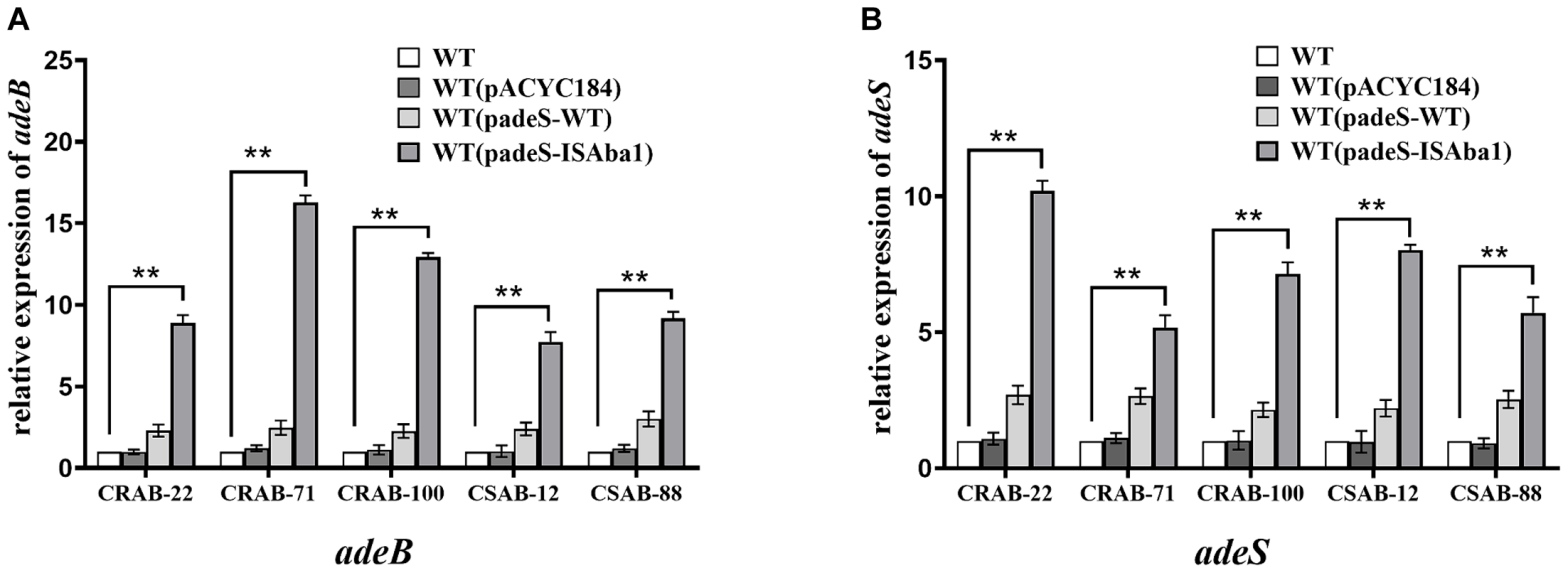
Expression level of *adeB* and *adeS* in strains transformed with padeS-ISAba1. Relative expression of *adeB* **(A)** and *adeS* **(B)** were assessed by qRT-PCR analysis. The housekeeping gene, *rpoB*, was used as an endogenous reference gene. The wildtype strain was used as the reference strain (expression = 1.0). All qRT-PCR experiments were carried out in triplicate. ** *p* < 0.05. WT: wildtype strain. WT (pACYC184): wildtype strain transformed with pACYC184. WT (padeS-WT): wildtype strain transformed with the padeS-WT. WT (padeS-ISAba1): wildtype strain transformed with the padeS-ISAba1.

The results described above revealed significant differences in the gene mutations observed in the three groups of resistant strains. The IS*Aba1* insertion in *adeS* was identified in 66.7% (6/ 9) of strains in the only ERV-resistant and both ERV- and TGC-resistant groups, while the IS*Aba1* insertion in *adeN* was found in 53.3% (8/15) of strains in the only TGC-resistant group. These findings suggest that the mechanisms of *A. baumannii* resistance to ERV and TGC may differ. To evaluate the impact of the IS*Aba1* insertion in *adeS* on ERV resistance, *adeS* with the IS*Aba1* insertion was cloned into the expression plasmid, pACYC184, and transformed into five strains that were sensitive to both ERV and TGC. Changes in the ERV and TGC MICs were then measured in the constructed strains. Strains transformed with padeS-ISAba1 exhibited an 8–16x increase in ERV MIC and a 4–16x increase in TGC MIC. All strains became resistant to ERV by acquiring padeS-ISAba1, and 80% (4/5) of the strains became resistant to TGC. No significant change in ERV and TGC MIC was found in strains transformed with padeS-WT or pACYC184 (Table 5). This result suggests that the IS*Aba1* insertion in *adeS* can confer resistance to ERV and TGC in *A. baumannii*. Expression levels of *adeB* and *adeS* in the transformed strains were further confirmed by qRT-PCR. The expressions of *adeB* and *adeS* were significantly upregulated in strains transformed with padeS-ISAba1, while the expression of them in strains transformed with padeSWT or pACYC184 showed no significant change (Figures 2A,B). This finding indicates that the IS*Aba1* insertion in *adeS* can enhance the expression of the efflux pump genes, *adeABC,* and its regulators, *adeRS*.

**Table 5:** Effect of padeS-ISAba1 transformation in susceptible strains on the ERV and TGC MICs.

Isolate	ERV MIC (mg/L)				TGC MIC (mg/L)			
	Wildtype strain^a^	Transform with pACYC184^b^	Transform with padeS-WT^c^	Transform with padeS-ISAba1^d^	Wildtype strain	Transform with pACYC184	Transform with padeS-WT	Transform with padeS-ISAba1
CRAB-22	1	1	2	8	2	2	4	16
CRAB-71	0.5	1	1	8	1	2	2	4
CRAB-100	1	1	2	16	1	1	2	16
CSAB-12	1	2	2	8	2	2	2	8
CSAB-88	0.5	0.5	1	8	1	2	2	16

a Wildtype strain: ERV- and TGC-susceptible *A. baumannii* isolate.

b Transform with pACYC184: Wildtype strain transformed with pACYC184.

c Transform with padeS-WT: Wildtype strain transformed with padeS-WT.

d Transform with padeS-ISAba1: Wildtype strain transformed with padeS-ISAba1.

To assess the role of the IS*Aba1* insertion in *adeN* on TGC resistance, the wildtype *adeN* was integrated into pACYC184 and transformed into five TGC-resistant strains with the IS*Aba1* insertion in *adeN*. The TGC and ERV MICs were then measured in the transformed strains. The TGC MIC of the complementary strains decreased by 4–16x after transformation with padeN-WT. Most (4/5, 80%) of the complementary strains had restored TGC sensitivity, while there was no significant change in ERV MIC for the complementary strains (Table 6). This result suggests that the expression of wildtype *adeN* can decrease the TGC MIC in resistant strains, and the disruption of *adeN* with the IS*Aba1* insertion confers TGC resistance. The expression of *adeJ* was significantly downregulated in all complementary strains transformed with padeN-WT (Supplementary Figure S2), indicating that the expression of wildtype *adeN* in strains with the IS*Aba1* insertion in *adeN* can repress *adeIJK* transcription.

**Table 6:** Effect of padeN-WT transformation in *adeN* mutant strains on the ERV and TGC MICs.

Isolate	TGC MIC (mg/L)			ERV MIC (mg/L)		
	Wildtype strain^a^	Transform with pACYC184^b^	Transform with padeN-WT^c^	Wildtype strain	Transform with pACYC184	Transform with padeN-WT
CRAB-35	16	32	1	2	2	2
CRAB-61	32	32	4	2	2	2
CRAB-158	16	16	2	1	1	0.5
CSAB-79	8	8	2	0.12	0.25	0.25
CSAB-166	8	8	1	2	2	1

a Wildtype strain: TGC-resistant strain with ISAba1 insertion in adeN.

b Transform with pACYC184: Wildtype strain transformed with pACYC184.

c Transform with padeN-WT: Wildtype strain transformed with padeN-WT.

## 4 Discussion

*A. baumannii* is a common pathogen associated with nosocomial infections that have posed a serious threat to public health for decades. CRAB is resistant to almost all commonly used antibiotics, leaving few available treatment options. TGC is one of the primary drugs used alone or in combination to treat CRAB infections, however, TGC resistance has gradually increased in recent years (Jo and Ko, 2021). ERV, a new-generation tetracycline, is shown to confer excellent antimicrobial activity against CRAB. Previous studies have compared the *in vitro* activity of ERV and TGC (Liu et al., 2022), while the different resistance mechanisms of these drugs against *A. baumannii* have not been explained. The current study sought to compare the characteristics and mechanisms of ERV and TGC resistance in *A. baumannii* to inform the clinical use of ERV in the treatment of CRAB infections.

CRAB exhibited an alarmingly high resistance rate to traditional tetracyclines, including doxycycline and minocycline (Xiong et al., 2023). ERV and TGC have been proven to overcome the main resistance mechanisms of tetracyclines, such as tetracycline-specific efflux Tet(A) and tetracycline resistance ribosomal protection protein Tet(M), which effectively elevated their antimicrobial activity to CRAB (Grossman et al., 2012; Yaghoubi et al., 2022). In this study, both ERV and TGC exhibited good antibacterial activity against CRAB, with MIC_90_ values of 1 mg/L and 2 mg/L, respectively, which was consistent with previous studies (Scheetz et al., 2007; Cruz-López et al., 2022). The ERV MIC_50_ and MIC_90_ of CRAB and CSAB isolates were 2x lower than those of TGC. Significantly fewer strains were resistant to ERV alone (*n* = 2) than to TGC alone (*n* = 15). Moreover, among strains that were resistant to both drugs, the ERV MIC was lower than the TGC MIC, indicating that ERV is a more effective drug against *A. baumannii*. Besides, high-dose regimens are required when TGC is used as monotherapy due to its low bioavailability (Cai et al., 2016). ERV had two unique changes compared to TGC structure, which greatly improved its bioavailability and antibacterial activity (Alosaimy et al., 2022). The MIC_50_ and MIC_90_ for ERV were higher in the CRAB than in the CSAB isolates, which was similar to the findings of Livermore et al. on carbapenem-resistant *Klebsiella pneumoniae* (Livermore et al., 2016). These data together suggest that the sensitivity of ERV is lower in carbapenem-resistant than in carbapenem-sensitive strains.

CRAB strains resistant to TGC and other tetracyclines primarily belong to the ST208 lineage in China (Deng et al., 2014; Jiang et al., 2019). In the current study, 77.8% (7/9) of the ERV-resistant strains were ST208 sequence types, suggesting that ST208 was also the major prevalent clone in ERV-resistant strain. Importantly, ERV- and/or TGC-resistant *A. baumannii* strains were primarily found to infect individuals >60 years of age and originate from the ICU, where patients are often immunocompromised. Strains resistant to both ERV and TGC may pose a serious clinical threat. While polymyxin B still exhibits antibacterial activity against ERV- and/or TGC-resistant CRAB isolates, this antibiotic is associated with severe nephrotoxicity and is not suitable for elderly patients with renal dysfunction, further limiting treatment options. Thus, there is a critical need to enhance the prevention and control of ERV and TGC-resistant strains in clinical practice. ERV- and/or TGC-resistant CSAB strains had high rates of resistance to minocycline and doxycycline, indicating the presence of cross-resistance among clinical *A. baumannii* strains. These data highlight the importance of regular monitoring and detection to prevent bacterial cross-resistance and treatment failure.

Overexpression of RND efflux pumps and mutations in their regulatory genes are the main mechanisms of *A. baumannii* resistance to TGC (Salehi et al., 2021). To determine the role of the RND efflux pump and its regulators in ERV- and/or TGC-resistant strains, the effect of EPIs on the ERV and TGC MICs was assessed. ERV and TGC MICs were significantly reduced in resistant strains following the addition of EPIs, suggesting that expression of the efflux pump may be associated with ERV and TGC resistance. Meanwhile, significant differences in genetic mutations and resistance gene expressions were identified among the three groups of resistant strains. The IS*Aba1* insertion in *adeS* was commonly found in the only ERV-resistant group and the both ERV- and TGC-resistant group, while the IS*Aba1*insertion in *adeN* was common in the only TGC-resistant group. The expressions of *adeABC* and *adeRS* were significantly increased in the only ERV-resistant group and in the both ERV- and TGC-resistant group, while *adeABC* and *adeIJK* expressions were significantly increased and *adeN* expression was significantly decreased in the only TGC-resistant group. Sun et al. found that an IS*Aba1* insertion in the *adeS* gene enhanced expression of the N-terminal truncated AdeS protein and further activated AdeR, leading to overexpression of *adeABC* and *A. baumannii* resistance to TGC (Sun et al., 2012). Lucaßen et al. found that the IS*Aba1* insertion could disrupt the function of AdeN, resulting in overexpression of *adeIJK* and contributing to TGC resistance in *A. baumannii* (Lucaßen et al., 2021a). Differences in gene mutations and expressions in the three groups of resistant strains identified in the current study indicate that the resistance mechanisms of ERV and TGC in *A. baumannii* may be various. Other resistance mechanisms apart from RND-type efflux pumps on ERV and TGC resistance have been reported previously. Wang et al. found that plasmid-mediated *tet*(X5) gene confers resistance to TGC and ERV in a clinical *A. baumannii* isolate (Wang et al., 2019). Hua et al. demonstrated that mutations in the genes of *adeS, rpoB* and *rrf* are associated with TGC resistance in *A. baumannii* (Hua et al., 2021). Other mutations in *rpsJ*, *tet(A), tetR,* and *soxR* were also reported to be associated with TGC resistance (Beabout et al., 2015; Linkevicius et al., 2016; Li et al., 2017; Zhang et al., 2021). However, few of the aforementioned resistance mechanisms were identified in the resistant strains of our study.

Functional validation experiments were performed to confirm the roles of IS*Aba1* insertion in *adeS* on ERV resistance and IS*Aba1* insertion in *adeN* on TGC resistance. Strains transformed with padeS-ISAba1 significantly increased the MICs of ERV and TGC and upregulated the expressions of AdeABC and AdeRS. These results suggest that the IS*Aba1* insertion in *adeS* promotes high AdeABC expression and contributes to both ERV and TGC resistance, similar to the TGC resistance mechanism reported by Sun et al. (2012). Previous studies have shown that ISA*ba1* insertion in *adeS* was rarely found in TGC resistance (Lucaßen et al., 2021a), but it is the primary mechanism conferring resistance to ERV in current study. Moreover, the TGC MICs of all *adeN* mutant strains complemented with padeN-WT were reduced to sensitive or intermediate levels, and the expression of *adeJ* in all complementary strains was significantly downregulated. This finding suggests that the expression of wildtype *adeN* can restore the TGC sensitivity of resistant strains by repressing *adeIJK* and the disruption of *adeN* by the IS*Aba1* insertion can increase AdeIJK expression and lead to TGC resistance, a result consistent with those of Rosenfeld et al. (2012). Notably, TGC resistant strains with IS*Aba1* insertion in *adeN* remained sensitive to ERV in our study and the ERV MIC did not show significant change in complementary strains transformed with padeN-WT. This suggests that ERV susceptibility is not affected by the IS*Aba1* insertion in *adeN*.

In conclusion, ERV exhibited excellent *in vitro* activity against both CRAB and CSAB and surpassed the efficacy of TGC. A major sequence type, ST208, was found in ERV- and/or TGC-resistant strains. Moreover, the IS*Aba1* insertion in *adeS* was shown to play a critical role in ERV resistance in *A. baumannii*, while the TGC resistance was mainly associated with the IS*Aba1* insertion in *adeN*.

## Data Availability

The original contributions presented in the study are included in the article/Supplementary material, further inquiries can be directed to the corresponding authors.
