# Supplementary material for: Comparison of antimicrobial activities and resistance mechanisms of eravacycline and tigecycline against clinical Acinetobacter baumannii isolates in China
Source: Front Microbiol. 2024 Sep 24;15:1417237. doi: 10.3389/fmicb.2024.1417237 (PMC11458409; doi:10.3389/fmicb.2024.1417237)
Supplement: Supplementary file 1 [file Data_Sheet_1.docx]

Supplementary Material

**Comparison of Antimicrobial Activities and Resistance Mechanisms of Eravacycline and Tigecycline Against** **Clinical *Acinetobacter baumannii* Isolates in China**

**Xiandi Chen^1^**†**, Yitan Li^1^**†**, Yingzhuo Lin^1^**†**, Yingyi Guo^2^, Guohua He^1^, Xiaohu Wang^1^, Mingzhen Wang^1^, Jianbo Xu^1^, Mingdong Song^1^, Xixi Tan^1*^, Chao Zhuo^2*^, Zhiwei Lin^1*^**

* Correspondence: Zhiwei Lin: 422156321@qq.com, Chao Zhuo: chao_sheep@263.net and Xixi Tan: 468212255@qq.com

1. **Supplementary Data**
2. **Supplementary Figures and Tables**

## Supplementary Figures


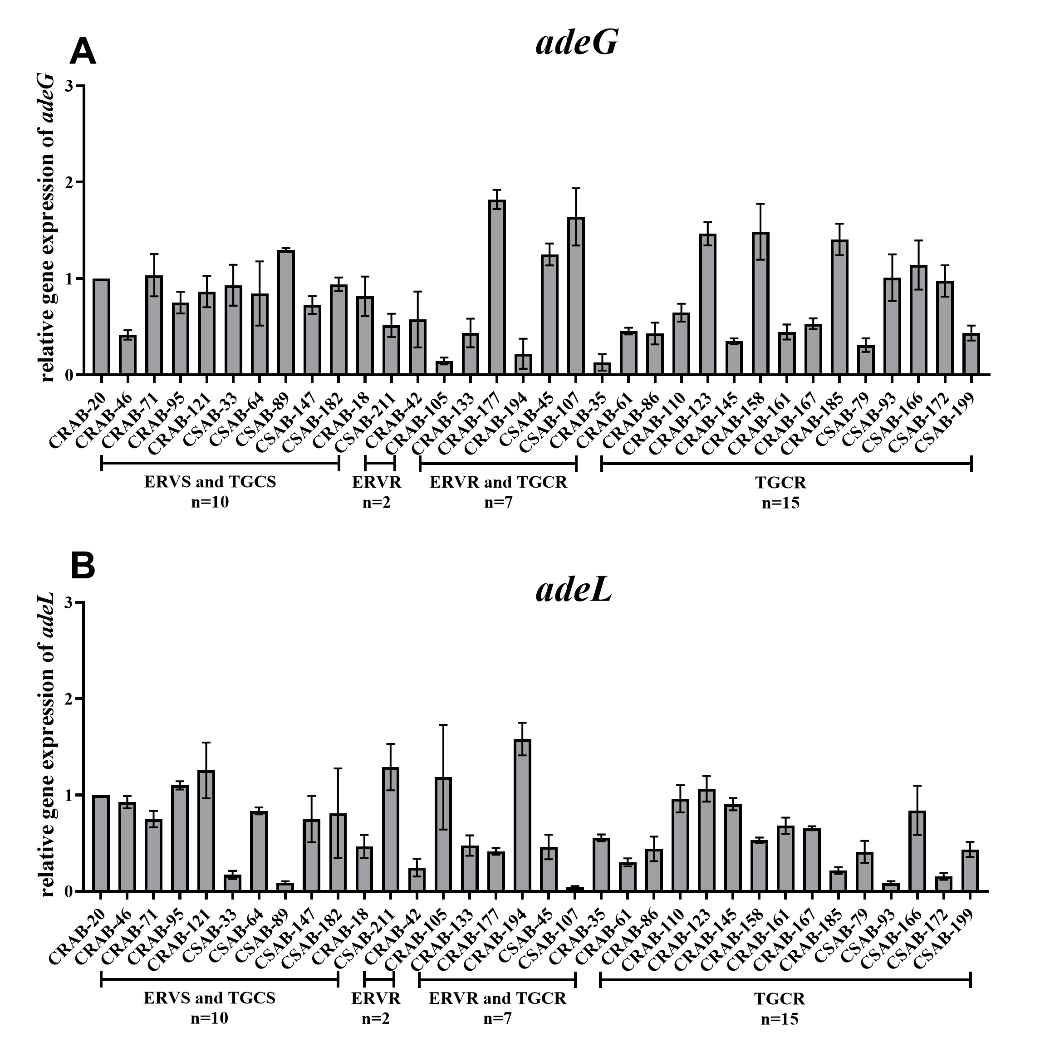


**Supplementary Figure 1.** Comparison of the relative expressions of efflux pump genes in ERV- and/or TGC-resistant isolates. Relative expression of *adeG* (A) and *adeL* (B) were assessed by qRT-PCR analysis. The housekeeping gene, *rpoB*, was used as the endogenous reference gene. CRAB-20 was used as the reference strain (expression = 1.0). All qRT-PCRs were carried out in triplicate. ∗∗*P* <0.05. ERVS and TGCS: strains sensitive to both ERV and TGC; ERVR: strains only resistant to ERV; ERVR and TGCR: strains resistant to both ERV and TGC; TGCR: strains only resistant to TGC.


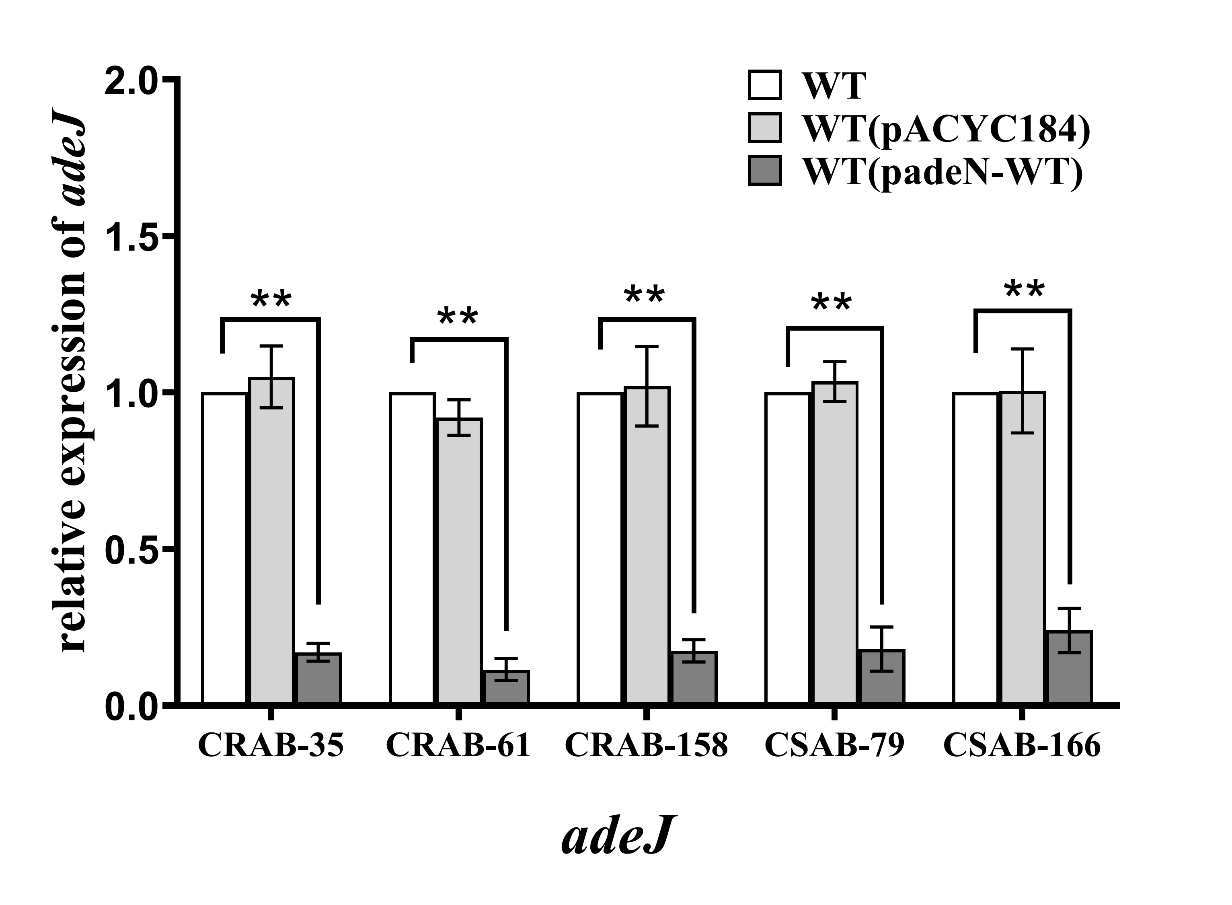


**Supplementary Figure 2.** Transcriptional level of *adeJ* in strains transformed with padeN-WT. Relative expression of *adeJ* were assessed by qRT-PCR analysis. The housekeeping gene, *rpoB*, was used as an endogenous reference gene. The wildtype strain was used as the reference strain (expression = 1.0). All qRT-PCR experiments were carried out in triplicate. ** *P* <0.05. WT: wildtype strain. WT (pACYC184): wildtype strain transformed with pACYC184. WT (padeN-WT): wildtype strain transformed with the padeN-WT.

## Supplementary Tables

**Supplementary Table 1.** Primers used for the Oxford scheme of MLST determination in *A. baumannii*.

| **Target gene** | **Primer** | **Primer sequence (5'to3')** | **Amplicon**  **size (bp)** | **Source** |
| --- | --- | --- | --- | --- |
| *gltA^a^* | gltA-F | AATTTACAGTGGCACATTAGGTCCC | 722 | (Bartual et al., 2005) |
|  | gltA-R | GCAGAGATACCAGCAGAGATACACG |  |  |
| *gyrB* | gyrB-F | TGAAGGCGGCTTATCTGAGT | 594 | (Bartual et al., 2005) |
|  | gyrB-R | GCTGGGTCTTTTTCCTGACA |  |  |
| *gdhB* | gdhB-SF | ACCACATGCTTTGTTATG | 774 | (Bartual et al., 2005) |
|  | gdhB-SR | GTTGGCGTATGTTGTGC |  |  |
| *recA* | recA-F | CCTGAATCTTCYGGTAAAAC | 425 | (Bartual et al., 2005) |
|  | recA-R | GTTTCTGGGCTGCCAAACATTAC |  |  |
| *cpn60* | cpn60-F | GGTGCTCAACTTGTTCGTGA | 640 | (Bartual et al., 2005) |
|  | cpn60-R | CACCGAAACCAGGAGCTTTA |  |  |
| *gpi* | gpi-F | GAAATTTCCGGAGCTCACAA | 456 | (Bartual et al., 2005) |
|  | gpi-R | TCAGGAGCAATACCCCACTC |  |  |
| *rpoD* | rpoD-F | ACCCGTGAAGGTGAAATCAG | 672 | (Bartual et al., 2005) |
|  | rpoD-R | TTCAGCTGGAGCTTTAGCAAT |  |  |

^a^*gltA*: citrate synthase; *gyrB*: DNA gyrase subunit B; *gdhB*: glucose dehydrogenase B; *recA*: homologous recombination factor; *cpn60*: 60-kDa chaperonin; *gpi*: glucose-6-phosphate isomerase; *rpoD*: RNA polymerase sigma factor.

**Supplementary Table 2.** Primers used for amplification of resistance genes in ERV- and/or TGC-resistant strains.

| **Target gene** | **Primer** | **Primer sequence (5'-3')** | **Amplicon**  **size (bp)** | **Source** |
| --- | --- | --- | --- | --- |
| Carbapenemase resistance genes | | | | |
| *bla*_KPC_ | KPC-F | CGTCTAGTTCTGCTGTCTTG | 798 | (Poirel et al., 2011) |
|  | KPC-R | CTTGTCATCCTTGTTAGGCG |  |  |
| *bla*_IMP_ | IMP-F | CTACCGCAGCAGAGTCTTTG | 587 | (Turton et al., 2004) |
|  | IMP-R | AACCAGTTTTGCCTTACCAT |  |  |
| *bla*_VIM_ | VIM-F | AGTGGTGAGTATCCGACAG | 261 | (Turton et al., 2004) |
|  | VIM-R | ATGAAAGTGCGTGGAGAC |  |  |
| *bla*_SIM_ | SIM-F | TACAAGGGATTCGGCATCG | 570 | (Poirel et al., 2011) |
|  | SIM-R | TAATGGCCTGTTCCCATGTG |  |  |
| *bla*_NDM-1_ | NDM1-F | TGCCGAGCGACTTGGCCTTG | 379 | (Ghazawi et al., 2012) |
|  | NDM1-R | ACCGATGACCAGACCGCCCA |  |  |
| *bla*_OXA-51_ | OXA51-F | TAATGCTTTGATCGGCCTTG | 353 | (Longjam et al., 2023) |
|  | OXA51-R | TGGATTGCACTTCATCTTGG |  |  |
| *bla*_OXA-23_ | OXA23-F | GATCGGATTGGAGAACCAGA | 501 | (Woodford et al., 2006) |
|  | OXA23-R | ATTTCTGACCGCATTTCCAT |  |  |
| *bla*_OXA-24_ | OXA24-F | GGTTAGTTGGCCCCCTTAAA | 246 | (Woodford et al., 2006) |
|  | OXA24-R | AGTTGAGCGAAAAGGGGATT |  |  |
| *bla*_OXA-48_ | OXA48-F | GCGTGGTTAAGGATGAACAC | 438 | (Poirel et al., 2011) |
|  | OXA48-R | CATCAAGTTCAACCCAACCG |  |  |
| *bla*_OXA-58_ | OXA58-F | AAGTATTGGGGCTTGTGCTG | 599 | (Woodford et al., 2006) |
|  | OXA58-R | CCCCTCTGCGCTCTACATAC |  |  |

**Supplementary Table 3.** Primers used for amplification of efflux pumps regulator, and other genes associated with ERV and TGC resistance in *A. baumannii* apart from efflux pump mechanisms.

| **Target gene** | **Primer** | **Primer sequence (5'-3')** | **Amplicon size (bp)** | **Source** |
| --- | --- | --- | --- | --- |
| *adeS* | adeS-F | GCGCTGCGGTTACAGCTTATAT | 1387 | This study |
|  | adeS-R | GAACCGTAGATAGCCATGTGAGT |  |  |
| *adeR* | adeR-F | GAGCTTAAGCTAATCCAGCC | 991 | This study |
|  | adeR-R | AAGTGTGGAGTAAGTGTGGAG |  |  |
| *adeL* | adeL-F | AAGATGGCAGACAGTGCAAACT | 1325 | This study |
|  | adeL-R | AAGCAATGATCGGTATTGACG |  |  |
| *adeN* | adeN-F | ACAATTATCTGTTGTTGGCTGG | 807 | This study |
|  | adeN-R | ACATACCAATGACCATCGTT |  |  |
| *rpsJ* | rpsJ-F | ACAGTCGTGGCATCGATCACG | 668 | This study |
|  | rpsJ-R | AGTTACGCGAGACTCGCGACGT |  |  |
| *tet*(X5) | tet(X5)-F | GTGGACCCGTTGGACTGACT | 1066 | This study |
|  | tet(X5)-R | TCGAGGCTGAACATTTCCGT |  |  |
| *tet(A)* | tet(A)-F | ATGTCCACCAACTTATCAGTG | 1176 | This study |
|  | tet(A)-R | GTTCCACGTTGTTATAGAAGC |  |  |
| *tetR* | tetR-F | CAGTGATAAAGTGTCAAGC | 643 | This study |
|  | tetR-R | AACGTTCCTGACAACGAGC |  |  |
| *rrf* | rrf-F | CGCTTCGGCATTAAATAAGACC | 615 | This study |
|  | rrf-R | GGCGTTAACGATAATGAATTCGC |  |  |
| *rpoB* | rpoB-F | GCATTTGTAAACAGTGGTGGCC | 701 | This study |
|  | rpoB-R | ACGACGGTCGATACGTACGT |  |  |
| *soxR* | soxR-F | GCATGATGAGCGCAAAGC | 925 | This study |
|  | soxR-R | CTAGCAGGATTTGGTGAACC |  |  |

**Supplementary Table 4.** Primers for qRT-PCR in this study.

| **Target gene** | **Primer^a^** | **Primer sequence (5'-3')** | **Amplicon size (bp)** | |
| --- | --- | --- | --- | --- |
| *rpoB* | qrpoB-F | CAGAAGAGAAGAACAAGTTAT | 103 |  |
|  | qrpoB-R | CGTGAAGAAGCATTAGTAG |  |  |
| *adeB* | qadeB-F | GGAATAAGGCACCACAACAAT | 75 |  |
|  | qadeB-R | CGAAGTTAGGAATACCAGCAATAC |  |  |
| *adeS* | qadeS-F | TTGCTGGTTCTCTACTAAG | 156 |  |
|  | qadeS-R | ACGCCTATAACGATATTACA |  |  |
| *adeG* | qadeG-F | GTTAATATCTGCTGATGTGTAA | 91 |  |
|  | qadeG-R | GGTGCCATTATCTTCATTG |  |  |
| *adeL* | qadeL-F | AATATGGCGAACCTACCT | 169 |  |
|  | qadeL-R | ATATAAGCATCACCGTCATT |  |  |
| *adeJ* | qadeJ-F | AACTACAACAGCATAGAG | 143 |  |
|  | qadeJ-R | AAGAACCAAGCAATAATATC |  |  |
| *adeN* | qadeN-F | CCATAATCATTCGCCATTCAA | 114 |  |
|  | qadeN-R | CTCAAGCCTTACTCATATCTCA |  |  |

^a^Primers for qRT-PCR were designed by Beacon designer software 8.14.

**Supplementary Table 5.** Bacterial strains used in functional verification experiment.

| **Name** | **Description^a^** | **Source** |
| --- | --- | --- |
| **Bacterial Strains** | | |
| *A. baumannii* | | |
| CRAB-22 | both ERV and TGC sensitive strains | People’s Hospital of Yangjiang |
| CRAB-77 | both ERV and TGC sensitive strains | People’s Hospital of Yangjiang |
| CRAB-100 | both ERV and TGC sensitive strains | People’s Hospital of Yangjiang |
| CSAB-12 | both ERV and TGC sensitive strains | People’s Hospital of Yangjiang |
| CSAB-88 | both ERV and TGC sensitive strains | People’s Hospital of Yangjiang |
| CRAB-22 (padeS-ISAba1^a^) | CRAB-22 introduced with plasmid padeS-ISAba1 | this study |
| CRAB-77 (padeS-ISAba1) | CRAB-77 introduced with plasmid padeS-ISAba1 | this study |
| CRAB-100 (padeS-ISAba1) | CRAB-100 introduced with plasmid padeS-ISAba1 | this study |
| CSAB-12 (padeS-ISAba1) | CSAB-12 introduced with plasmid padeS-ISAba1 | this study |
| CSAB-88 (padeS-ISAba1) | CSAB-88 introduced with plasmid padeS-ISAba1 | this study |
| CRAB-22 (padeS-WT^b^) | CRAB-22 introduced with plasmid padeSWT | this study |
| CRAB-77 (padeS-WT) | CRAB-77 introduced with plasmid padeSWT | this study |
| CRAB-100 (padeS-WT) | CRAB-100 introduced with plasmid padeSWT | this study |
| CSAB-12 (padeS-WT) | CSAB-12 introduced with plasmid padeSWT | this study |
| CSAB-88 (padeS-WT) | CSAB-88 introduced with plasmid padeSWT | this study |
| CRAB-22 (pACYC184) | CRAB-22 introduced with plasmid pACYC184 | this study |
| CRAB-77 (pACYC184) | CRAB-77 introduced with plasmid pACYC184 | this study |
| CRAB-100 (pACYC184) | CRAB-100 introduced with plasmid pACYC184 | this study |
| CSAB-12 (pACYC184) | CSAB-12 introduced with plasmid pACYC184 | this study |
| CSAB-88 (pACYC184) | CSAB-88 introduced with plasmid pACYC184 | this study |
| CRAB-35 | TGC resistant strains with IS*Aba1* insertion in *adeN* | People’s Hospital of Yangjiang |
| CRAB-61 | TGC resistant strains with IS*Aba1* insertion in *adeN* | People’s Hospital of Yangjiang |
| CRAB-158 | TGC resistant strains with IS*Aba1* insertion in *adeN* | People’s Hospital of Yangjiang |
| CSAB-79 | TGC resistant strains with IS*Aba1* insertion in *adeN* | People’s Hospital of Yangjiang |
| CSAB-166 | TGC resistant strains with IS*Aba1* insertion in *adeN* | People’s Hospital of Yangjiang |
| CRAB-35(padeN-WT^c^) | CRAB-35 introduced with plasmid padeNWT | this study |
| CRAB-61(padeN-WT) | CRAB-61 introduced with plasmid padeNWT | this study |
| CRAB-158(padeN-WT) | CRAB-158 introduced with plasmid padeNWT | this study |
| CSAB-79(padeN-WT) | CSAB-79 introduced with plasmid padeNWT | this study |
| CSAB-166(padeN-WT) | CSAB-166 introduced with plasmid padeNWT | this study |
| CRAB-35(pACYC184) | CRAB-35 introduced with plasmid padeNWT | this study |
| CRAB-61(pACYC184) | CRAB-61 introduced with plasmid padeNWT | this study |
| CRAB-158(pACYC184) | CRAB-158 introduced with plasmid padeNWT | this study |
| CSAB-79(pACYC184) | CSAB-79 introduced with plasmid padeNWT | this study |
| CSAB-166(pACYC184) | CSAB-166 introduced with plasmid padeNWT | this study |
| *E. coli* | | |
| DH5α | *supE44 △lacU169 hsdR17 recA1 endA1 gyrA96 thi-1 relA1* | Invitrogen |
| **Plasmids** | | |
| pACYC184 | Medium copy number vector, p15A ori, *Cm*, *Tc* | (Lin et al., 2018) |
| padeS-ISAba1 | pACYC184 inserted with *adeS* with IS*Aba1* insertion | this study |
| padeSWT | pACYC184 inserted with wild type *adeS* | this study |
| padeNWT | pACYC184 inserted with wild type *adeN* | this study |

^a^*adeS* with IS*Aba1* insertion was cloned into expression plasmid pACYC184.

^b^Wild type of *adeS* gene was cloned into expression plasmid pACYC184.

^c^Wild type of *adeN* gene was cloned into expression plasmid pACYC184.

**Supplementary Table 6.** Primers used for functional verification experiment.

| **Primers** | **Sequences（5’-3’）** | **Product**  **Length (bp)** | **Underline**  **(enzyme site)** |
| --- | --- | --- | --- |
| **Construction of the recombinant plasmid** | | | |
| padeS-ISAba1-F | CGCGGATCCGACTAGATAATCCCCTAGCTG | 2489 | BamHI |
| padeS-ISAba1-R | CCATCGATACAGCTTATATGTTAGGTGTC |  | ClaI |
| padeN-WT-F | CGCGGATCCGCTGATGACGATGATGAGGAC | 1117 | BamHI |
| padeN-WT-R | CCATCGATAGAGCCATGTAGCTACTCCAT |  | ClaI |
| **Verification of the recombinant plasmids** | | | |
| IDpadeS-ISAba1-F | ATCCTTGAAGCTGTCCCTGAT | 989 |  |
| IDpadeS-ISAba1-R | TGGCTAACCAAATCCAGTCTA |  |  |
| IDpadeN-WT-F | AGTGATCGAAGTTAGGCTGGT | 1603 |  |
| IDpadeN-WT-R | AGAGGAATGATCAGGAGATGT |  |  |

**Supplementary Table 7.** The sample sources of 492 *A. baumannii* isolates.

| **Source** | **Strains (n, %)** | | **Total (n, %)** |
| --- | --- | --- | --- |
|  | **CRAB (n = 253)** | **CASB (n =239)** |  |
| Sputum | 154 (60.9) | 65 (27.2) | 219 (44.5) |
| Wound secretion | 62 (24.5) | 59 (24.7) | 121 (24.6) |
| Urine | 10 (4.0) | 57 (23.8) | 67 (13.6) |
| Blood | 9 (3.6) | 35 (14.6) | 44 (8.9) |
| Bronchoalveolar lavage fluid | 6 (2.4) | 9 (3.8) | 15 (3.0) |
| Ascites | 6 (2.4) | 8 (3.3) | 14 (2.8) |
| Cerebrospinal fluid | 2 (0.8) | 0 (0) | 2 (0.4) |
| Abscess | 3 (1.2) | 3 (1.3) | 6 (1.2) |
| Bile | 1 (0.4) | 2 (0.8) | 3 (0.6) |
| Pleural effusion | 0 (0) | 1 (0.4) | 1 (0.2) |

**Supplementary Table 8.** The antimicrobial activity to common antibiotics among the different groups of resistant isolates.

| **Strains (n=24)** | **resistance (n, %)** | | | | | | |
| --- | --- | --- | --- | --- | --- | --- | --- |
|  | polymyxin B**^a^** | levofloxacin | minocycline | amikacin | gentamicin | doxycycline | cefepime |
| CRAB^b^ (n=16) | | | | | | | |
| Only ERV-resistant group (n=1) | 0 (0) | 1 (100) | 1 (100) | 1 (100) | 1 (100) | 1 (100) | 1 (100) |
| Both ERV- and TGC-resistant group (n=5) | 0 (0) | 5 (100) | 5 (100) | 5 (100) | 5 (100) | 5 (100) | 4 (80) |
| Only TGC-resistant group (n=10) | 1 (10) | 10 (100) | 10 (100) | 10 (100) | 10 (100) | 10 (100) | 10 (100) |
| CSAB (n=8) | | | | | | | |
| Only ERV-resistant group (n=1) | 0 (0) | 0 (0) | 1 (100) | 0 (0) | 0 (0) | 1 (100) | 0 (0) |
| Both ERV- and TGC-resistant group (n=2) | 0 (0) | 1 (50) | 2 (100) | 0 (0) | 0 (0) | 2 (100) | 0 (0) |
| Only TGC-resistant group (n=5) | 1 (20) | 0(0) | 5 (100) | 0 (0) | 1 (20) | 5 (100) | 2 (40) |

^a^CRAB: Carbapenem-resistant *A. baumannii*, CSAB: Carbapene-susceptible *A. baumannii*.

**Supplementary Table 9.** Mutations of other genes associated with ERV and TGC resistance in *A. baumannii* apart from efflux pump mechanisms.

| **Strains (n = 24)** | **Mutation** | | | | | | |
| --- | --- | --- | --- | --- | --- | --- | --- |
|  | ***rpsJ*** | ***tet(A)*** | ***tetR*** | ***tet*(X5)** | ***rrf*** | ***rpoB*** | ***soxR*** |
| **Only ERV-resistant group (n = 2)** | | | | | |  |  |
| CRAB-18 | W^a^ | W | W | /^b^ | W | W | W |
| CSAB-211 | W | W | W | / | W | W | W |
| **Both ERV- and TGC-resistant group (n = 7)** | | | | | |  |  |
| CRAB-42 | W | W | W | / | W | W | W |
| CRAB-105 | V57I | W | W | / | W | W | W |
| CRAB-133 | W | W | W | / | W | W | W |
| CRAB-177 | W | W | W | / | W | W | W |
| CRAB-194 | W | M247S | W | / | W | W | W |
| CSAB-45 | W | W | W | / | W | W | W |
| CSAB-107 | W | W | W | / | W | W | W |
| **Only TGC-resistant group (n = 15)** | | | | | |  |  |
| CRAB-35 | W | W | W | / | W | W | W |
| CRAB-61 | W | W | W | / | W | W | W |
| CRAB-86 | W | W | N27P | / | W | W | W |
| CRAB-110 | W | W | W | / | W | W | W |
| CRAB-123 | W | W | W | / | W | W | W |
| CRAB-145 | W | W | W | / | W | W | W |
| CRAB-158 | W | W | W | / | W | W | W |
| CRAB-161 | W | W | W | / | W | W | W |
| CRAB-167 | W | W | W | / | W | W | W |
| CRAB-185 | W | W | W | / | W | W | W |
| CSAB-79 | W | W | W | / | W | W | W |
| CSAB-93 | W | W | W | / | W | W | W |
| CSAB-166 | W | W | W | / | W | W | W |
| CSAB-172 | W | W | W | / | W | W | W |
| CSAB-199 | W | W | W | / | W | W | W |

^a^"W" indicates that no mutations were detected in the gene.

^b^"/" indicates that *tet*(X5) was not detected in all strains.

1. **References**

Bartual, S.G., Seifert, H., Hippler, C., Luzon, M.A., Wisplinghoff, H., and Rodríguez-Valera, F. (2005). Development of a multilocus sequence typing scheme for characterization of clinical isolates of Acinetobacter baumannii. *J Clin Microbiol* 43(9)**,** 4382-4390. doi: 10.1128/jcm.43.9.4382-4390.2005.

Ghazawi, A., Sonnevend, A., Bonnin, R.A., Poirel, L., Nordmann, P., Hashmey, R., et al. (2012). NDM-2 carbapenemase-producing Acinetobacter baumannii in the United Arab Emirates. *Clin Microbiol Infect* 18(2)**,** E34-36. doi: 10.1111/j.1469-0691.2011.03726.x.

Lin, Z., Cai, X., Chen, M., Ye, L., Wu, Y., Wang, X., et al. (2018). Virulence and Stress Responses of Shigella flexneri Regulated by PhoP/PhoQ. *Frontiers in Microbiology* 8. doi: 10.3389/fmicb.2017.02689.

Longjam, L.A., Tsering, D.C., and Das, D. (2023). Molecular Characterization of Class A-ESBLs, Class B-MBLs, Class C-AmpC, and Class D-OXA Carbapenemases in MDR Acinetobacter baumannii Clinical Isolates in a Tertiary Care Hospital, West Bengal, India. Cureus 15(8), e43656. doi: 10.7759/cureus.43656.

Poirel, L., Walsh, T.R., Cuvillier, V., and Nordmann, P. (2011). Multiplex PCR for detection of acquired carbapenemase genes. *Diagn Microbiol Infect Dis* 70(1)**,** 119-123. doi: 10.1016/j.diagmicrobio.2010.12.002.

Turton, J.F., Kaufmann, M.E., Warner, M., Coelho, J., Dijkshoorn, L., van der Reijden, T., et al. (2004). A prevalent, multiresistant clone of Acinetobacter baumannii in Southeast England. *J Hosp Infect* 58(3)**,** 170-179. doi: 10.1016/j.jhin.2004.05.011.

Woodford, N., Ellington, M.J., Coelho, J.M., Turton, J.F., Ward, M.E., Brown, S., et al. (2006). Multiplex PCR for genes encoding prevalent OXA carbapenemases in Acinetobacter spp. *Int J Antimicrob Agents* 27(4)**,** 351-353. doi: 10.1016/j.ijantimicag.2006.01.004.
